# Supplementary figures and images for: Freshwater-adapted sea bass Dicentrarchus labrax feeding frequency impact in a lettuce Lactuca sativa aquaponics system
Source: PeerJ. 2021 Jun 3;9:e11522. doi: 10.7717/peerj.11522 (PMC8180194; doi:10.7717/peerj.11522)

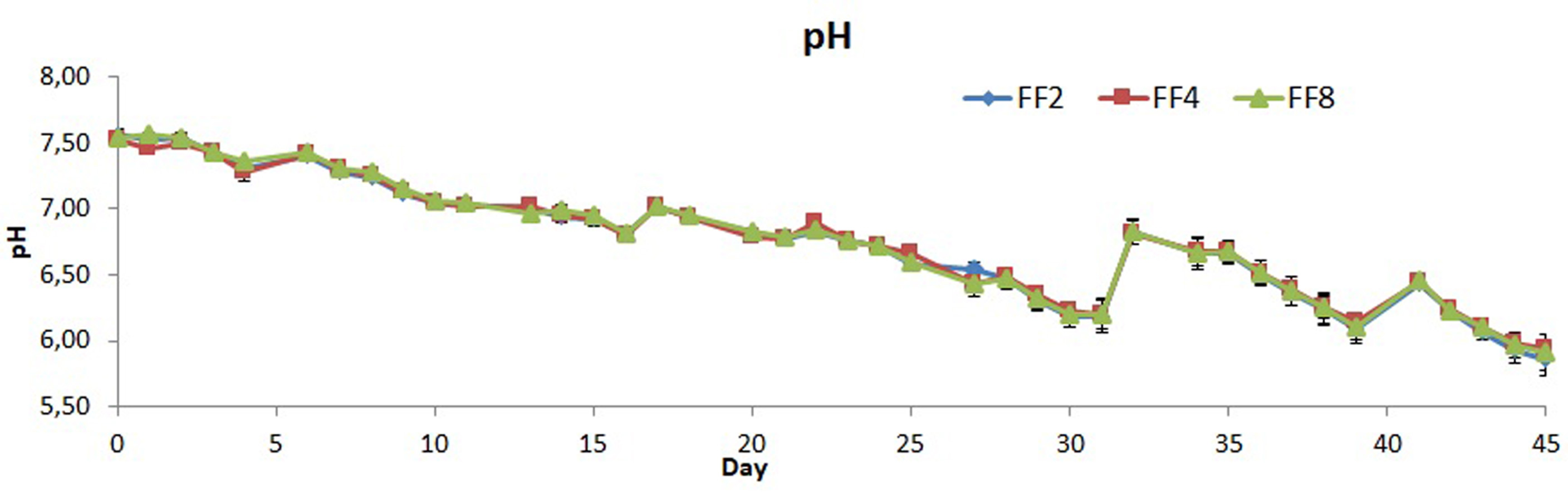

Supplement: Supplemental Information 1 [file peerj-09-11522-s001.jpg]

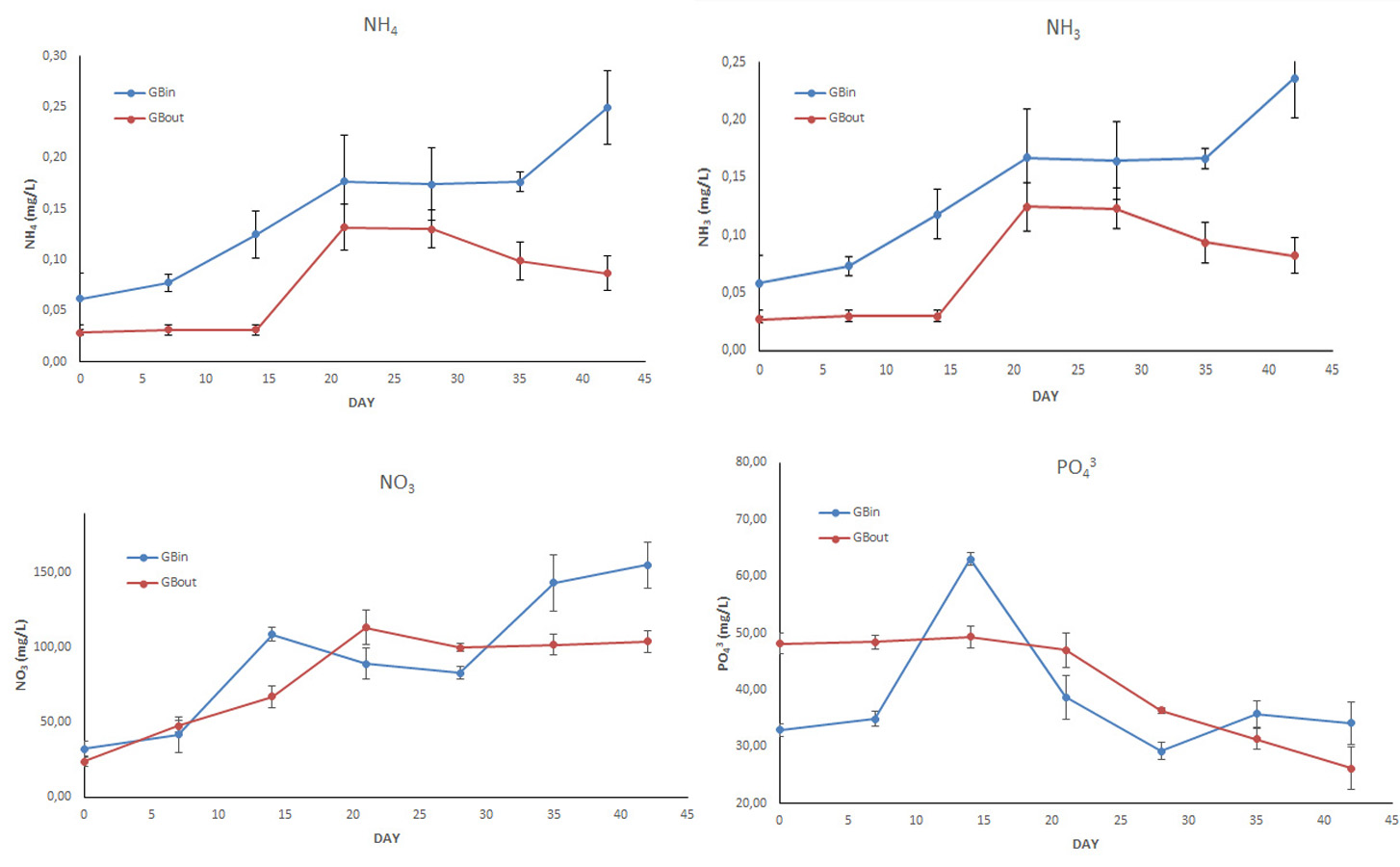

Supplement: Supplemental Information 2 — Mean A) NH4, B) NH3, C) NO3 and D) PO43 fluctuation at the water inlet point (GBin) and at the exit point (GBout) of the hydroponic cultivation tank for all three systems during the study period. [file peerj-09-11522-s002.jpg]
